# Unveiling hole trapping and surface dynamics of NiO nanoparticles†
†Electronic supplementary information (ESI) available. See DOI: 10.1039/c7sc03442c


**DOI:** 10.1039/c7sc03442c

**Published:** 2017-10-25

**Authors:** Luca D'Amario, Jens Föhlinger, Gerrit Boschloo, Leif Hammarström

**Affiliations:** a Department of Chemistry – Ångström Laboratory , Uppsala University , Box 523 , 751 20 Uppsala , Sweden . Email: leif.hammarstrom@kemi.uu.se ; Fax: +46 18 471 6844 ; Tel: +46 18 471 3648

## Abstract

The research effort in mesoporous p-type semiconductors is increasing due to their potential application in photoelectrochemical energy conversion devices. In this paper an electron–hole pair is created by band-gap excitation of NiO nanoparticles and the dynamics of the electron and the hole is followed until their recombination. By spectroscopic characterization it was found that surface Ni^3+^ states work as traps for both electrons and holes. The trapped electron was assigned to a Ni^2+^ state and the trapped hole to a “Ni^4+^” state positioned close to the valence band edge. The recombination kinetics of these traps was studied and related with the concept of hole relaxation suggested before. The time scale of the hole relaxation was found to be in the order of tens of ns. Finally the spectroscopic evidence of this relaxation is presented in a sensitized film.

## Abstract 2

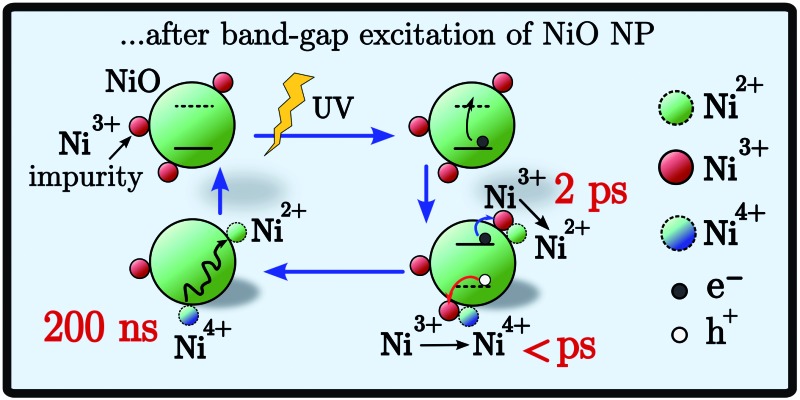
Mesoporous NiO is used as p-type material in photoelectrochemical energy conversion devices. The presence of two kinds of hole traps can affect device performance. Here, after band-gap excitation, the relaxation of the hole into two different traps was observed and characterized.

## Introduction

In the last fifty years the electronic properties of nickel oxide (NiO) have been the focus of theoretical and experimental studies and are still under debate.^1^–^6^ NiO, in its pure form, is a 3.5 eV indirect band gap semiconductor showing charge-transfer/Mott–Hubbard insulating behaviour.^7^,^8^ It has been used as semiconductor material in a range of potential solar energy conversion applications such as hole transport layer in organic photovoltaic cells, water splitting cells, dye-sensitized solar fuel cells (DSSFCs) and p-type dye-sensitized solar cells (p-DSCs).^9^–^15^ Often in these devices NiO is in the form of nanostructured crystals which are sintered together to obtain a mesoporous film with a large active area.^16^,^17^ The surface of the crystals plays a central role in the activity of the semiconductor. This means that most of the past studies measuring bulk properties might not be representative of those observed in the large active surface material. NiO surface is not stoichiometric and hosts Ni^3+^ sites together with the Ni^2+^ ones.^18^–^21^ In DSSFCs and p-DSCs the surface of the NiO crystals is covered with catalysts and/or photosensitizers.^13^,^14^,^22^,^23^ In these devices the photosensitizer injects a hole in the valence band (VB) of NiO upon photo excitation. This creates a charge separation between the hole in the nanoparticle and the electron in the dye or in the catalyst. The conversion efficiency of these devices is strictly related to the capacity of the dye/NiO (or catalyst/NiO) interface to maintain long-lived charge separation.^24^–^26^ Despite many years of research in DSC, the photocathodes built using NiO as semiconductor are still not efficient (record of photo-conversion efficiency 1.3%).^27^ The reason of the poor performance of NiO based dye sensitized devices has been mainly attributed to recombination losses.^14^,^24^,^25^,^28^–^30^ Namely, the hole, after being injected, can react with the electron in the reduced dye or the reduced part of the redox couple or reduced catalyst. This wastes all the energy accumulated in the initial charge separation. Many attempts to reduce the recombination can be found in the literature. NiO has been doped with various metals^31^–^36^ and the dye has been furnished with an electron acceptor or spacers or different binding groups.^37^–^47^ Lately our group used procedures that reduced the high valence Ni impurities in the nanoparticles, which may be the main channel for the hole recombination.^24^ In fact, Ni^3+^ surface states can oxidise and trap a hole to “Ni^4+^”, where “Ni^4+^” indicates a mixed valence state§
§The oxidation states of Ni higher than 2 in NiO are probably mixed-valence states that we for simplicity denote Ni^3+^ and “Ni^4+^”. of Ni higher than 3, possibly hybridized with the valence band, which has not been well characterised yet.^20^,^48^–^51^

After being trapped, the “Ni^4+^” holes can react with the electrolyte or the reduced dye. This mechanism has been just hypothesized and it has been supported only by indirect evidences.^24^,^25^ So far, no direct experimental proof of such hole trapping has been found since there is no spectral characterization of such species. A better understanding of the hole traps in NiO is needed to give a direction to the effort in NiO applied research. Moreover, a study on the reactivity of Ni^3+^ sites would improve our understanding of the devices where NiO is used either as an oxygen evolving catalyst for water oxidation or as a photocatalyst for water reduction. The reactivity of the Ni^3+^ states could play an important role in the catalytic activity in both applications.^10^,^11^,^50^–^54^

Recently, our group was able to assign the characteristic spectra to both the high valence Ni states present in NiO nanoparticles, Ni^3+^ and “Ni^4+^”.^24^ This now gives the possibility to use transient absorption spectroscopy (TAS) to investigate the nature and dynamics of the hole trapping in mesoporous NiO. In this paper band gap excitation has been used to generate an electron–hole pair that we followed from the creation to the final recombination, passing through the steps of electron and hole trapping. This work aims to investigate the dynamics of the trapping process and find the spectral evidence of the trapped hole to be used in the characterization of NiO photocathodes.

## Results and discussion

Mesoporous NiO films were prepared using sol–gel doctor blading method. This kind of preparation is commonly used in p-type dye sensitized solar cells (DSCs) and solar fuel devices (DSSFDs).^13^,^55^ The absorption spectrum of the NiO film prepared on a CaF_2_ window is reported in Fig. 3, the black curve. The fundamental absorption of NiO begins at around 370 nm. In the inset (a) of Fig. 3, a small absorption band is visible around 380 nm, which might be attributed to the direct excitation of an electron to a trap state. The excitation wavelengths used in this study with bare NiO are all equal to or lower than 355 nm, which leads to band gap excitation (BG ex.).

**Fig. 1:**
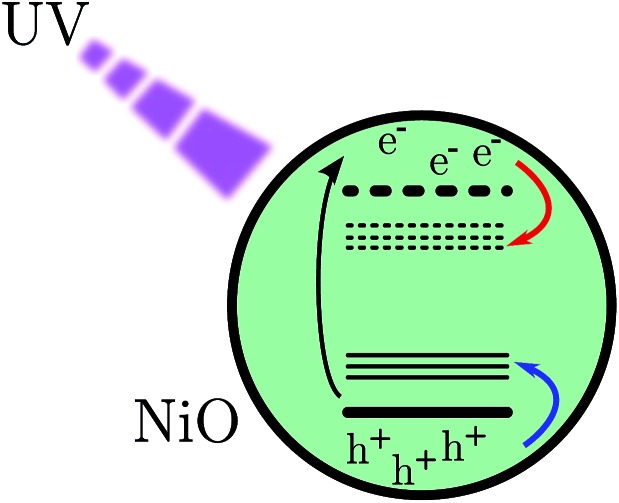
Scheme of band gap excitation. The red arrow indicates a movement of an electron and the blue one indicates the movement of a hole.

After BG excitation, promoting an electron from the valence band (VB) to the conduction band (CB), the free electron and free hole can be trapped by the intra gap states below the CB and above the VB, respectively (see Fig. 1). It should be noted that in the case of 355 nm the excitation is located at the bottom of the band gap absorption, which means that it brings an electron from the extreme edge of the valence band to the extreme edge of the conduction band.

**Fig. 2:**
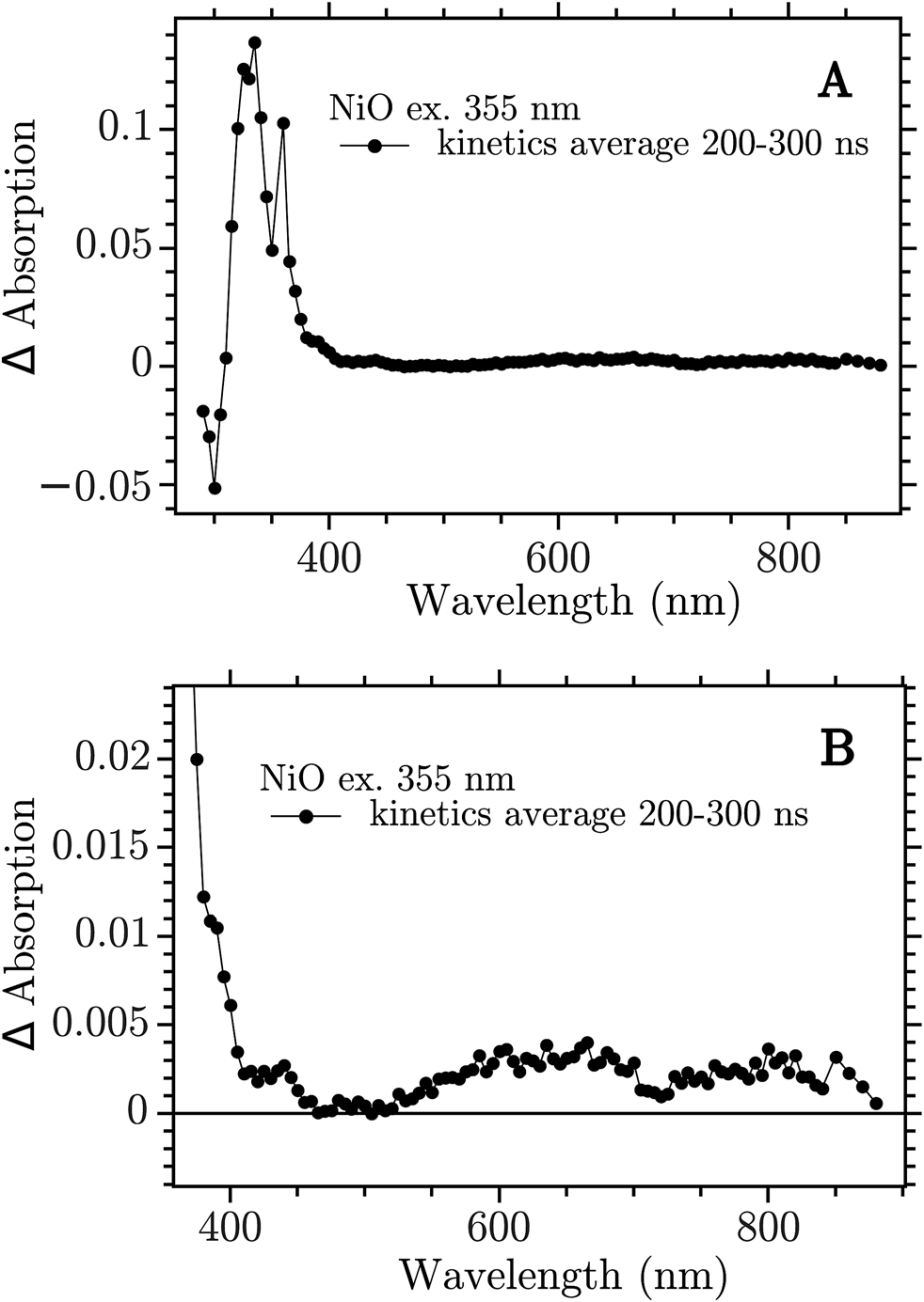
Transient absorption spectra at 200–300 ns after band-gap excitation of NiO on CaF_2_ windows. (A) Full spectrum; (B) 350–900 nm zoom.

The sample was excited with a 355 nm (12 ns) pulse and the transient absorption spectra (TA) were reconstructed by averaging of the monochromatic kinetic traces recorded each 10 nm in the interval 290–900 nm. The TA of the NiO film on CaF_2_ recorded 200–300 ns after excitation is reported in Fig. 2.

The spectrum showed in Fig. 2a is the full TA spectrum recorded from 290 nm to 900 nm. It shows a very strong absorption band between 310 and 360 nm and a pronounced bleach from 290 to 310 nm. In Fig. 2b a magnification of the visible region is reported. The spectrum in this region is more structured and it shows the “red end” of the UV band that dominates the TA in Fig. 2a, with a shoulder at around 400 nm, a smaller band at around 430 nm and two broad bands at 550–700 nm and 700–900 nm, respectively. This spectrum is due to the electron–hole pair created in the band gap excitation. The CaF_2_ substrate does not contribute to the signal, as proven in the ESI (Fig. S1†). To ensure that the spectrum obtained here derives from a BG transition the ns-TAS measurement was repeated with a shorter excitation wavelength, 266 nm. The two TA spectra show the same features, see Fig. S2 in the ESI.†

The 330 nm band decay kinetics is well fitted by a biexponential function with close-lying time constant: 2 μs (79%) and 7 μs (21%), see Fig. S10.† The bands at 430 and 650 nm do not show the same kinetics of the band in the UV but show halftimes of only 200–300 ns, see Fig. S11.† The kinetics of the visible bands fits well with a second order decay, see Fig. S13.† It seems that the UV band is not correlated with the spectral features in the visible.

In the following we show that the visible bands can be assigned to the creation of “Ni^4+^” states by hole trapping, while the TA in the UV region is due to heating of the NiO film by laser excitation. We also show evidence for electron trapping on the ps timescale, leading to reduction of Ni^3+^ states to Ni^2+^.

**Fig. 3:**
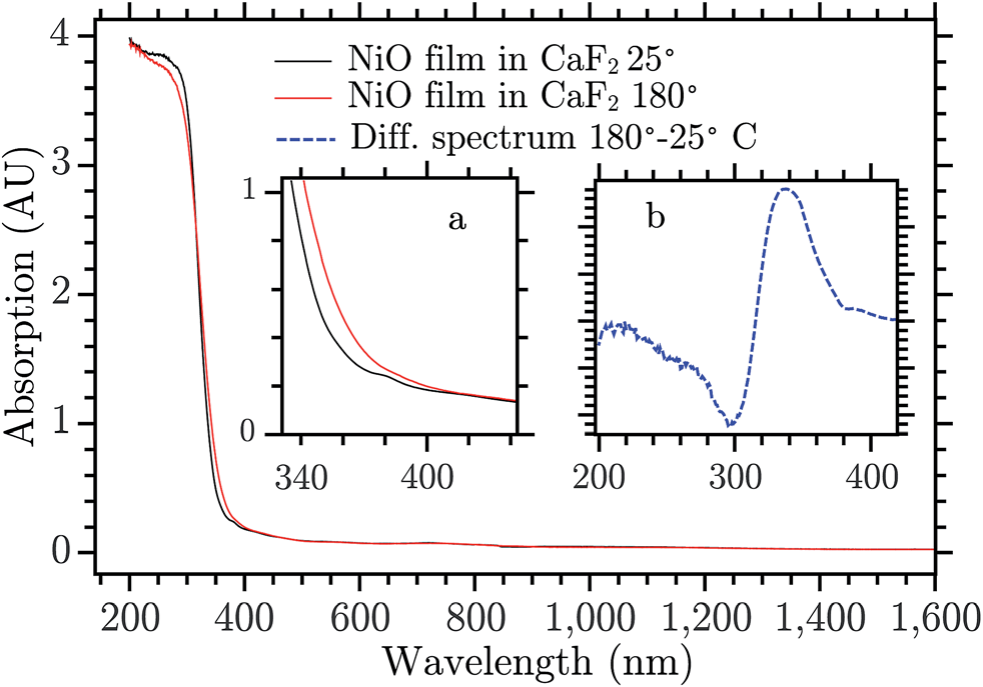
Absorption spectrum of NiO on CaF_2_ recorded at two temperatures 25 °C (black) and 180 °C (red). Inset (a) Enlargement in the 300–400 nm range. Inset (b) Spectral difference of the absorption spectrum of NiO at two temperatures (180° and 25 °C).

The 330 nm band seems to arise from a temperature change of the sample upon laser excitation. Thermal relaxation of the excited electron increases the temperature of the NiO film that changes its absorption spectrum. Fig. 3 shows the spectrum of NiO film recorded at two temperatures, room temperature (RT) and 180 °C, black and red curve, respectively.¶
¶The RT spectrum was obtained after preheating the NiO film to 200° to remove Ni^3+^ impurities.^24^ The spectra do not present differences except for the edge of the fundamental absorption. In the inset (b) the difference of the spectra recorded at the two temperatures is reported. The UV band of the TA spectrum is very similar to the absorption difference due to the temperature increase, they differ only by a small shift of the entire band towards the blue (for a direct comparison see Fig. S3, in the ESI†). The deviations can be due to the fact that the temperature difference spectrum was obtained under equilibrium conditions while the TA spectrum is presumably far from equilibrium. The UV TA band is therefore attributed to thermal relaxation. Its decay, in Fig. S10,† is thus attributed to the thermal dissipation. A rough estimate‖
‖The estimate was done by assuming that the entire energy of the pulse absorbed by the film is converted to heat raising the temperature of the film by *C*_p_ × Δ*T* (*C*_p_ heat capacitance). of the temperature locally and temporarily reached by the NiO immediately after the laser pulse is about 150 °C. There are many possible reasons why the temperature changes the absorption of a semiconductor, for example a shift of the band gap, change of the distribution of phonons in an indirect semiconductor, Burstein shift, modification of the DOS of the semiconductor, change in chemical composition, and change in reflectance. All these phenomena can change the absorption spectrum and the result seen here can be a combination of all them. No further investigation was done to understand the reason of this effect since it is beyond the scope of this work. The rest of the discussion will focus on the visible part of the spectrum.

**Fig. 4:**
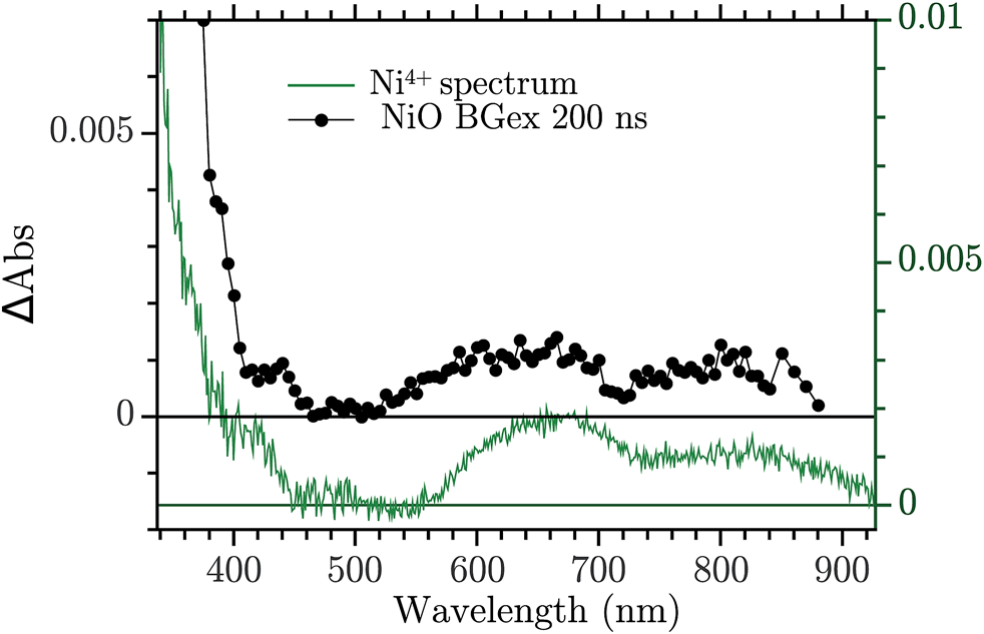
Comparison between the TA at 200 ns after BG excitation and the “Ni^4+^” characteristic spectrum from ref. 24.

The assignment of the Vis TA spectrum in Fig. 2b was done by comparing the TA to the characteristic spectra of the high valence Ni state present in NiO nanoparticles. These spectra, of Ni^3+^ and “Ni^4+^” states, have been recently published by our group.^24^ In Fig. 4, the characteristic spectrum of “Ni^4+^” (green) and the TA of NiO after BG excitation (black) are compared. The two spectra look remarkably similar, the “Ni^4+^” spectrum is only slightly shifted vertically with respect to the TA. It is now briefly recalled how the “Ni^4+^” and Ni^3+^ spectra were found. In our previous work the spectro-electro-chemistry (SEC) of mesoporous NiO was recorded across the entire range of potential where Ni^2+^ is oxidised to Ni^3+^ and Ni^3+^ to “Ni^4+^”. The spectra of Ni^3+^ and “Ni^4+^” were extracted by selecting the SEC spectrum at the potential of the oxidation of the species of interest. The potentials were chosen from an analysis of the DOS of NiO, measured previously.^31^ A closer look at Fig. 4 reveals that the TA is slightly distorted with respect to the SEC spectrum. This could be due to solvent or electrolyte effects that affects only the SEC.

**Fig. 5:**
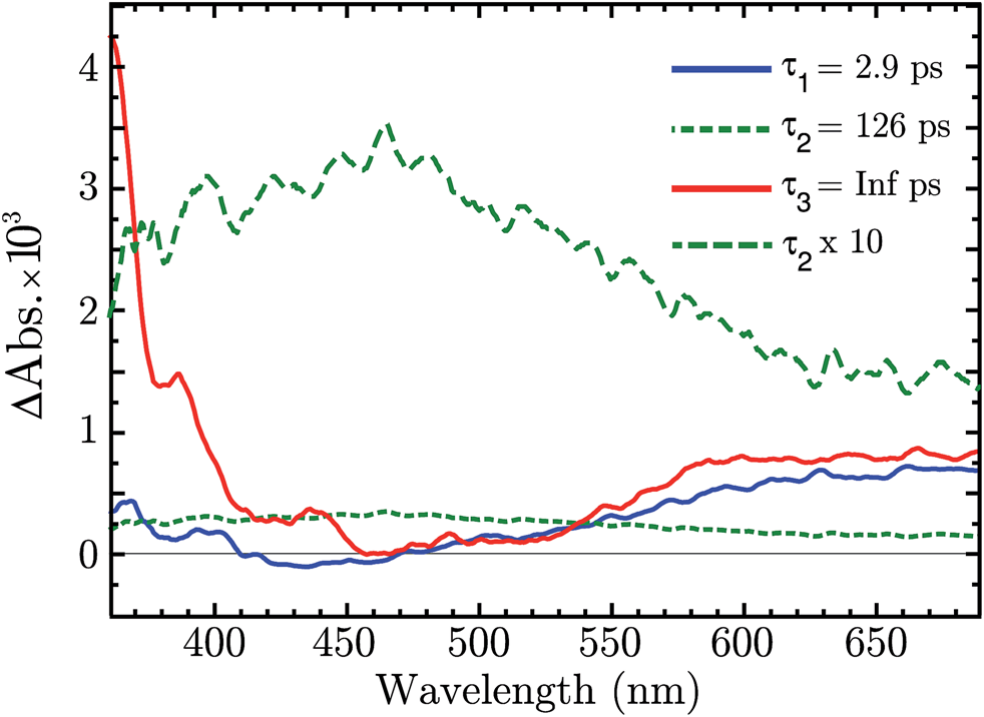
Decay associated spectra of the fs-TA of NiO after 330 nm excitation.

There is now the evidence that “Ni^4+^” is produced after BG excitation of NiO. This might be the result of the hole trapping from the VB to a Ni^3+^ state. Ultrafast TAS was performed to resolve the trapping process. The NiO was excited with 355 nm pulses (140 fs, 250 nJ per pulse, 1 kHz). The TA data on a 1–1900 ps time scale was globally fitted with a sum of three exponential function, with time constant *τ*_1_ = 2.9 ps, *τ*_2_ = 126 μs and *τ*_3_ = ∞. The amplitudes of each component as a function of probe wavelength give decay associated spectra (DAS), which are plotted in Fig. 5. The raw fs-TA spectra are reported in the ESI.†

The three DAS thus show the TA changes associated with each time constant. The blue spectrum, *τ*_1_, shows mainly a broad absorption band in the red that decays with *τ*_1_ = 2.9 ps, and a small absorption around 430 nm that rises with the same lifetime. The green spectrum, *τ*_2_, covers the entire visible range with a slightly higher absorption between 380 to 600 nm, which decays in 126 ps. The red spectrum, *τ*_3_, is the infinite component which has no corresponding rise in the DAS. As shown from the raw fs-TA spectra of Fig. S5,† the 0.5–2.6 ps spectra already present the features of the infinite component. Considering that *τ*_1_ DAS exhibits only small changes we deduce that the infinite component is present at all times (*t* < 0.5 ps) after the excitation.

**Fig. 6:**
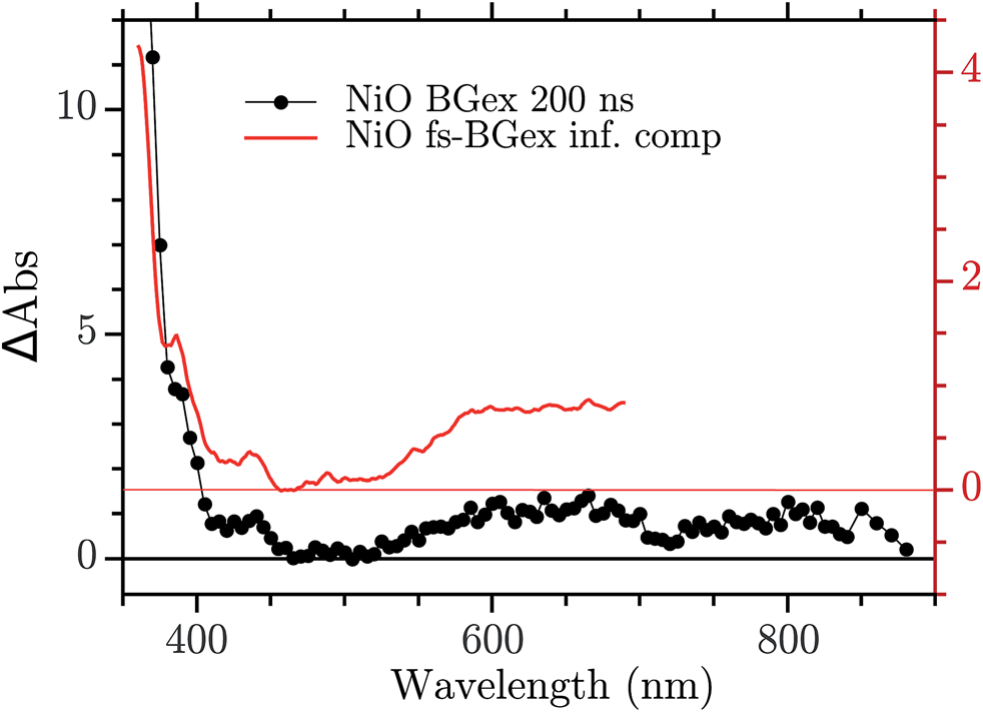
Comparison between the infinite component of the DAS in the fs–ns regime and the ns TA at 200 ns.

**Fig. 7:**
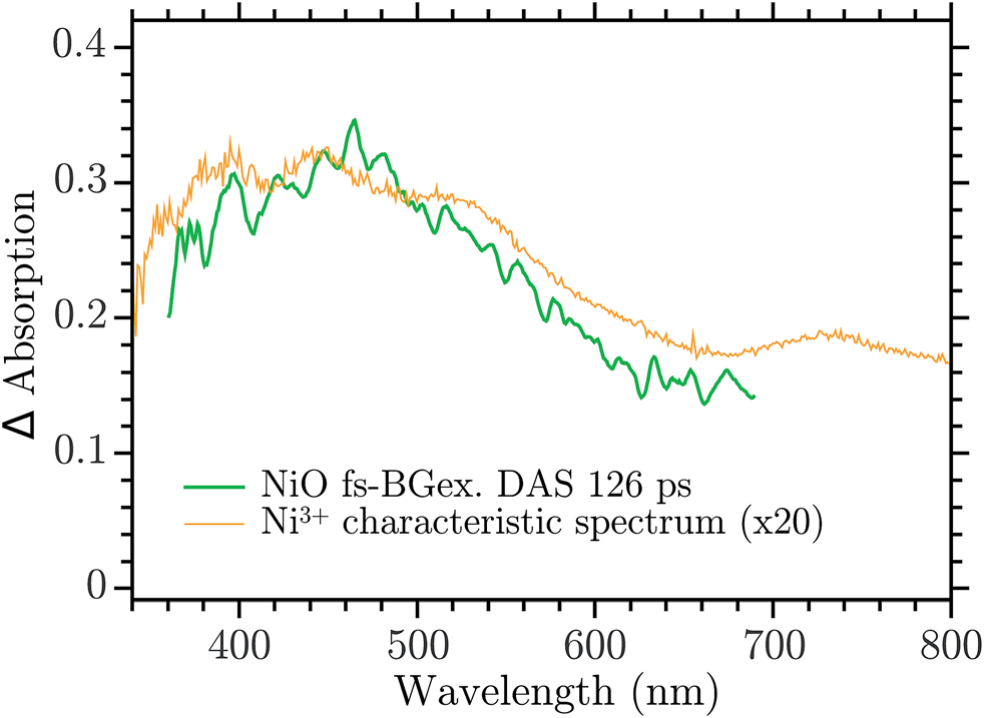
Comparison between the 126 ps component of the DAS in the Ni^3+^ characteristic spectrum found in ref. 24.

The infinite component in the fs-TA and the absorption seen in the ns-TA experiment are very similar, see Fig. 6. It seems that this species, which is thought to be “Ni^4+^”, is present from the very beginning of the observed transient kinetics. We think that this could correspond to the result of a very quick, sub-picosecond, hole trapping at the Ni^3+^ sites. This trapped hole survives until it recombines with the electron in the nanosecond regime. The electron signal we assign to the blue and green spectra in the DAS, Fig. 5. The blue spectrum shows a broad absorption in the red that, at this time-scale after a BG excitation, is normally attributed to the electron in the conduction band.^56^,^57^ It decays in 2.9 ps leading to the rise of a small absorption at 450 nm (note that a negative value of a DAS means an absorption increase). This amplitude can be attributed to the relaxation of electrons in shallow traps, in states close to the CB. In principle, the 2.9 ps spectrum could originate also from hole trapping but we exclude this eventuality since the hole trapping signal was already assigned to the infinite component. Moreover, the 2.9 ps component could contain also the signal changes from direct recombination of electron and trapped hole but this would be a minor pathway as the amplitude of the 2.9 ps component at around 400 nm is small. After the localization in the shallow states, the electrons eventually end up in deep traps that we believe are Ni^2+^ states due to a reduction of Ni^3+^ states. This statement is supported by the DAS of the *τ* = 126 ps component in Fig. 5 that shows the decay of an absorption that is now assigned to the Ni^3+^. Once again this assignment is based on the characteristic spectra found in ref. 24, see Fig. 7.

This assignment places the electron deep trap very close to the valence band. The reduction of Ni^3+^ to Ni^2+^ in NiO nanoparticles was found to occur at –100 mV *vs.* Ag/Ag^+^ thus just 400–500 mV above the valence band.^24^ In fact, the electron generated by BG excitation was found to be rather unreactive while normally an electron in a p-type semiconductor CB is considered a strong reducing agent. We performed BG excitation of a NiO film wetted by various solutions of electron acceptors (*e.g.* a saturated solution of methyl viologen) but none of them showed reactivity with the CB electron, see ESI.† The conclusion is that the electron in the CB is quickly deactivated by the deep Ni^2+^ traps and can not react any more with the acceptor molecules.

**Fig. 8:**
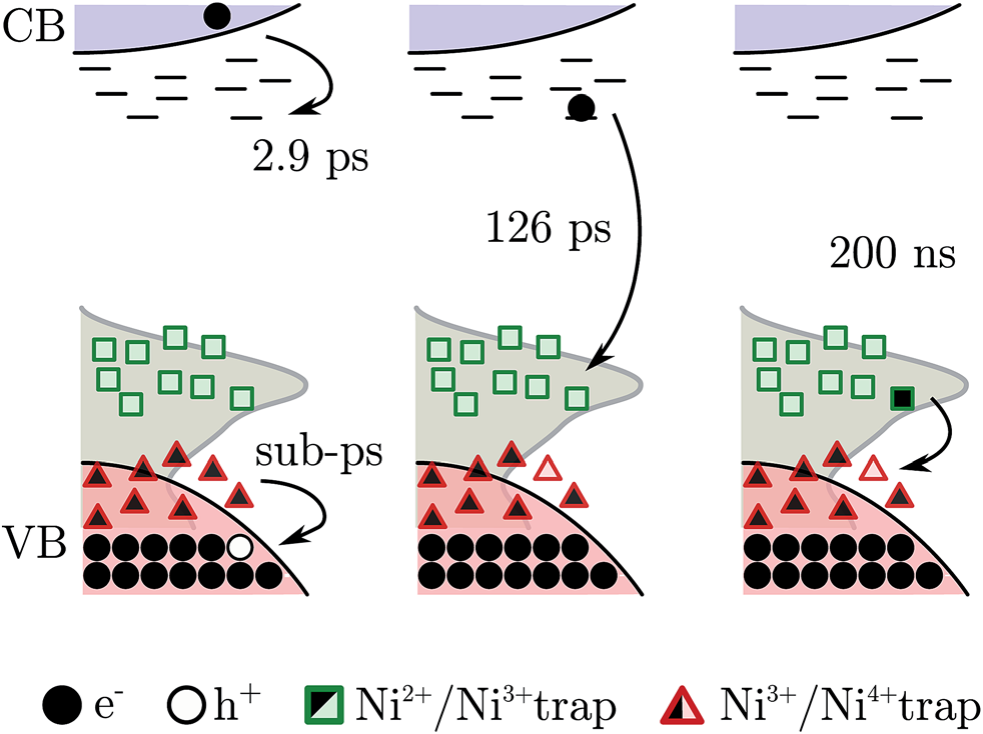
Scheme of the electron–hole dynamics after BG excitation. The pink area represents the VB, the light blue area shows the CB, the squares and triangles are the trap states, and the gray area gives the experimental reference of the DOS. Arrows indicate the movement of an electron.

Summarizing, the fs-TAS showed that after fs BG excitation the hole created in NiO nanoparticles is quickly trapped at a Ni^3+^ site that becomes a “Ni^4+^” site, see scheme in Fig. 8; this process will be called *hole shallow-trapping*. The electron instead is first trapped in a shallow trap and in a second step it falls into a deep trap, at a Ni^3+^ site turning Ni^2+^, process which we call *electron shallow-trapping* and *electron deep-trapping* respectively. The hole and electron traps, the “Ni^4+^” and Ni^2+^ states respectively, can react and close the cycle by giving back the two starting Ni^3+^ states, a process we denote *trap annihilation*.

It has been proven that Ni^3+^ is found on the surface of NiO as a result of a Ni^2+^ vacancy.^20^,^21^,^58^ Thus the carriers, trapped as “Ni^4+^” and Ni^2+^, might reside on the surface of the nanoparticles and need to move to react with each other. This is supported by the second order character of the annihilation reaction on a 100's of nanosecond time scale (Fig. S13,† discussed above). A second order kinetics has already been observed in electron–hole trap reactions and it is associated with a random walk of the trapped species on the semiconductor surface.^59^

The mechanism just described should be suppressed if the Ni^3+^ is removed from the NiO nanoparticle. Many ways to reduce the amount of Ni^3+^ in NiO can be found in literature. Recently our group reported a method that effectively reduces the Ni^3+^ states on the NiO surface by heating the film at 200 °C for two minutes.^24^ The fs-TAS BG excitation experiment was repeated upon Ni^3+^ removal, the result is shown in Fig. S4.† As expected, the signals due to the “Ni^4+^” and Ni^2+^ traps are completely suppressed. Only one component remains relevant, the decay of the electron in the CB (*τ*_1_ = 2.9 ps, in the non-annealed film), which still can occur. This test experiment supports the hypothesis that the electron and hole trapping occur on the surface of the nanoparticle.

The understanding of the hole dynamics is essential for possible applications of NiO mesoporous films in photoelectrochemical devices. In solar fuel and photovoltaic cells hole injection into the VB of NiO is photoinduced by a sensitizer. Keeping the hole far away from the NiO surface is crucial to avoid any kind of recombination with the reduced dye, *i.e.* avoid power losses. These results suggest that after injection the hole is trapped on a sub-picosecond timescale in a highly reactive “Ni^4+^” state sitting on the surface of NiO nanoparticle. This of course opens paths for rapid dye- and electrolyte-hole recombination. Moreover these results help understanding also the dynamics between different trap states that follow the initial hole trapping. We now know that a hole in NiO can be trapped in a “Ni^4+^” (oxidation of Ni^3+^) or a Ni^3+^ (oxidation of Ni^2+^) site. The “Ni^4+^” can be considered a shallow trap for holes since they are very close to the VB (0–100 mV above^24^,^31^). Since this vicinity to the VB, it is also possible that these “Ni^4+^” states are indeed part of the VB. This hypothesis is ruled out by the fact that in the heat treated sample “Ni^4+^” is not produced. The Ni^3+^ traps instead are located deeper in the band gap, about 300–500 mV above the VB. Thus, after the quick trapping, the hole can relax to deeper traps, if these are “empty”.**
**A empty trap state for a hole is a state that is filled with an electron. See the following reaction:

1 Ni_(h^+^)_^4+^ + Ni^2+^ → Ni^3+^ + Ni_(h^+^)_^3+^

here the (h^+^) subscript notation indicate a trapped hole. This process, that will be called *hole relaxation*, was invoked from our group in a work where two dye-hole recombination paths were observed and the presence of two kinds of holes (*i.e.* quickly and slowly recombining holes) was hypothesised.^28^ Here the direct evidence of the presence of two holes and the relaxation from one to another is found. In fact the *trap annihilation* process that it is observed here after BG excitation can be represented by the reaction:

2 Ni_(h^+^)_^4+^ + Ni_(e^–^)_^2+^ → Ni^3+^ + Ni^3+^

Reaction (1) and (2) can be considered the same reaction, they involve an electron transfer from a “Ni^4+^” to a Ni^2+^. The only difference is that reaction (2) benefits of an intrinsic charge neutralization missing in reaction (1). In reaction (2) the hole and the electron neutralize each other keeping the nanoparticle neutral without the help of an external neutralizing species. While in reaction (1) the hole does not benefit from the charge of an electron, thus it needs to be neutralized by an external species. Under device working condition it can be assumed that the injected hole is charge neutralized by lattice modification and by the electrolyte in contact with the nanoparticle. Thus the two reactions, the *trap annihilation* and the *hole relaxation*, can occur on similar timescales. The kinetics of both reactions is governed by the concentration of the species on the surface of NiO. In the *trap annihilation* one can assume that Ni_(h^+^)_^4+^ and Ni_(e^–^)_^2+^ have the same concentration since they are the product of the BG excitation. This agrees with the observation of second order kinetics of the *trap annihilation*. The hypothesis of second order kinetics is supported by the dependence of the rate on the excitation light intensity. The reaction half-life, *t*_1/2_, is shorter with higher light intensities, see S12 in ESI.† In the ESI† a more complete discussion of the *trap annihilation* kinetics is given. It was not possible to calculate a rate constant since data on the extinction coefficients and therefore concentrations are missing. An idea of the kinetics of this reaction can be given by the half-lifetime that, in the used range of excitation intensities, was between 100 and 300 ns.

**Fig. 9:**
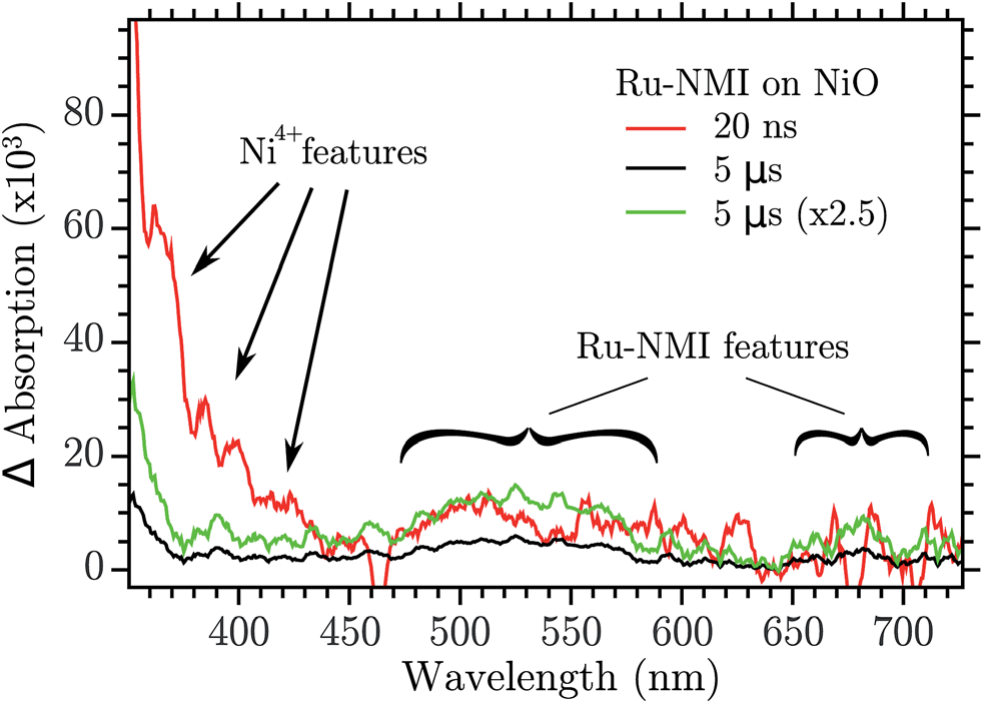
Transient absorption of Ru-NMI sensitized on NiO and excited at 460 nm. The green trace is the 5 μs trace normalized to the 20 ns one at 500 nm.

In ref. 28 it was hypothesized that two kinds of holes could recombine with very different kinetics with the electron in the reduced dye. This was based on the strongly biphasic charge recombination kinetics for dye-sensitized NiO, with one ∼15 ns component and one stretched component that decayed over 1 μs to 1 s time scale. It was also proposed that a relaxation between the two holes could transfer hole population from the quick to the slow recombining holes. The results presented here give direct evidence for this hole relaxation (eqn (1)), and also show that it involves a “Ni^4+^” hole that reacts with a Ni^2+^ state to produce a Ni^3+^ hole in tens of nanoseconds. This identifies the “Ni^4+^” trap as the hole showing fast recombination with reduced dyes, while the Ni^3+^ trap shows the slower recombination. The hole relaxation kinetics is proportional to the concentration of the donor and the acceptor state, namely the “Ni^4+^” and Ni^2+^. This means that if the Ni^2+^ surface states are few enough the kinetics is so slow that the “Ni^4+^” holes could be observed in a dye sensitized NiO nanoparticles by ns-TAS. TAS was performed with a NiO mesoporous film that was sensitized with a Ru-based dye, Ru-NMI,^45^ formula in the ESI.† The result is reported in Fig. 9.

After excitation of the metal to ligand charge transfer band, Ru-NMI injects a hole in the VB of NiO. The following recombination is relatively slow (>1 μs). The spectrum of the reduced state of the dye has a very characteristic spectrum with a band in the 500–570 nm region and another band around 700 nm. Two transient spectra are reported, one at 20 ns after excitation and another after 5 μs. The 20 ns spectrum shows the visible region features of the reduced Ru-NMI and, more importantly, the features of the “Ni^4+^” spectrum: the bands at 380, 400 and 430 nm. The spectrum recorded at 5 μs instead does not have the “Ni^4+^” features while it still shows the absorption of the dye, see the green spectrum in Fig. 9. The spectrum of “Ni^4+^” disappeared due to the hole relaxation. The relaxed hole then recombines more slowly with the reduced dye. These results confirm the model of relaxation–recombination of ref. 28.

## Conclusion

In this work transient absorption spectroscopy was used to analyse the trapping of electrons and holes generated by band gap excitation of NiO nanoparticle films. The analysis revealed that after a fs pulse the hole is trapped on a sub-picosecond time scale in a “Ni^4+^” state. The electron in the conduction band is firstly trapped in a shallow trap (*τ* ∼ 2.9 ps), then it falls in a deep trap very close to the valence band (*τ* ∼ 126 ps). Both the electron and hole traps are due to Ni^3+^ states that are located on the surface of NiO. The electron deep trap is then a Ni^2+^ state and the hole one is a “Ni^4+^” state. This assignment were made on the basis of our previously reported spectra of these species. Transient absorption spectroscopy was able to resolve also the trap annihilation (eqn (2)) that was useful to extract information on the hole relaxation between the “Ni^4+^” and Ni^3+^ traps. The two species responsible for the biphasic recombination kinetics previously found for dye-sensitized NiO were then assigned: “Ni^4+^” to the fast recombination phase (100 ns) and Ni^3+^ to the slow recombination phase (1 ms). Hole relaxation is then confirmed in NiO nanoparticle surface and it occurs in tens of ns. Finally the hole relaxation was directly probed in a dye sensitized system when NiO was sensitized with a Ru dye. The excitation produced firstly the reduced dye and the trap in the “Ni^4+^” state whereas after 200 ns the “Ni^4+^” signal disappeared while the reduced dye was still observable. This gives direct support to the proposal od “Ni^4+^” hole relaxation on a nanosecond time scale.

These results greatly aid the characterization of mesoporous NiO electrodes, with the spectral evidence of the two hole traps Ni^3+^ and “Ni^4+^”. In fact these spectra could be used in the future to understand which kind of hole are generated in the cells. Moreover the proof of a rapid (sub-picosecond) hole trapping into Ni_(h^+^)_^4+^ by Ni^3+^ sites on the surface of NiO gives an important warning and direction of where to look for better performance. Regarding dye sensitized devices the key factor is reducing the reactivity of the Ni_(h^+^)_^4+^ hole. This could be achieved in two ways. First, removing the Ni^3+^ sites from the surface of NiO by chemical reduction would avoid “Ni^4+^” formation. Second, retarding the electron recombination with Ni_(h^+^)_^4+^ to lifetimes longer than 100–200 ns would allow the relaxation to the Ni_(h^+^)_^3+^ hole for which recombination is much slower. Moreover these results could also be useful for understanding the activity of NiO when used as a catalyst, *i.e.* in a water splitting cell. As mentioned in the introduction, NiO can be used either as oxygen evolving catalyst as well as hydrogen evolving photocatalyst. In the first case the creation of a “Ni^4+^” species could play a role in the mechanism of water oxidation. On the same topic, these results could help in understanding the role of dopants, like Fe^3+^, that are effective in enhancing the catalytic performance of NiO. A doping site might need to compete with Ni^3+^ traps to be able to become active (*i.e.* host a hole). Instead, in the case of photocatalytic reduction, electrons are injected in the CB of NiO and then transferred to the electron acceptor. Ni^3+^ sites can trap the electrons, inactivating the catalyst.

In general this work proves that Ni^3+^ sites that are normally found in NiO nanoparticles are very active in trapping electrons and holes. It is our opinion that researcher working in NiO nanostructured materials should put a greater effort in the understanding of the reactivity of Ni^3+^ impurities.

## Experimental

### General

UV-Vis absorption was recorded by a Varian Cary 5000 spectrophotometer. NiO films were prepared on CaF_2_ UV-grade windows (1 inch diameter) that were ordered from Crystran. All chemicals were ordered by Sigma-Aldrich and used as received. NiO mesoporous films were prepared on CaF_2_ windows following a procedure already described elsewhere.^31^ The NiCl_2_ powder used for the preparation was 99.99% pure with 0.8 ppm Fe impurities. Bare NiO films on CaF_2_ were then used as taken from the oven with no further treatment. The sensitised films were obtained by immersing the NiO film into 0.5 mM Ru-NMI MeCN overnight, and then were rinsed by MeCN.

### Transient absorption measurements

*ns-TAS*. NiO band gap excitation was provided by frequency tripled Nd:YAG laser (Quantel, BrilliantB) that delivered 355 nm pulses of 7 ns duration which was QS-delay modulated to obtain pulse energies in the range 0.1–5 mJ per pulse. Analyzing light was supplied by a pulsed 150 W xenon lamp in a flash photolysis spectrometer (Applied Photophysics, LKS80). Light that passed through the sample was sent through a monochromator set to a bandwidth of 2 nm prior to reaching the 5 stage P928 photomultiplier tube (Hamamatsu). The signal was recorded using an Agilent Technologies Infiniium digital oscilloscope (600 MHz). Transient absorption trace were produced by the Applied Photophysics LKS software package by averaging 10–100 single traces. The analysis of the traces were performed by the open source software qtiplot and octave. The atmosphere of the chamber was controlled, when needed, by inflating dry nitrogen inside the sample chamber. *fs-TAS*. The femtosecond to picosecond TA set-up has been described previously.^60^ Shortly, 355 nm pump light was obtained by second harmonic generation of the 710 nm output of a optical parametric amplifier (TOPAS-white, Light Conversion) and directed on the sample. Pump energies were kept at around 250 nJ per pulse. Probe light was obtained from white light generation in a CaF_2_ crystal. The orientation of the polarization of pump and probe pulse was kept to 54.7° in order avoid anisotropy effects. Data fitting of the femtosecond to picosecond TA data was done using a lab written MATLAB (The MathWorks, Inc.) script. To obtain the lifetime of the different components, kinetic traces starting from 1 ps (chirpcorrected) at several wavelengths were fitted globally to a sum of exponential decays and an offset. The obtained lifetimes were fixed and used for fitting every wavelength to obtain the decay associated spectra (DAS) being the amplitude of the pre-exponential coefficients of the components at the respective wavelength.

## Conflicts of interest

The authors declare no competing financial interest.

## Supplementary Material

Supplementary information
