# Supplementary material for: Unveiling hole trapping and surface dynamics of NiO nanoparticles
Source: Chem Sci. 2017 Oct 25;9(1):223–30. doi: 10.1039/c7sc03442c (PMC5869301; doi:10.1039/c7sc03442c)
Supplement: Supplementary file 1 [file SC-009-C7SC03442C-s001.pdf]

Electronic Supplementary Material (ESI) for Chemical Science.
This journal is © The Royal Society of Chemistry 2017

Supporting information for: Unveiling hole trapping and surface dynamics of NiO nanoparticles.

Luca D'Amario,[†,‡] Jens Föhlinger,[†,‡] Gerrit Boschloo,[†,¶] and Leif Hammarström[*,†]

Department of Chemistry - Ångström Laboratory, Uppsala University, Box 523, 751 20 Uppsala, Sweden, , and

Center of Molecular Devices, Department of Chemistry, Royal Institute of Technology (KTH), 10044 Stockholm, Sweden

E-mail: leif.hammarstrom@kemi.uu.se

Phone: +46 (0)18 471 3648. Fax: +46 (0)18 471 6844

ns TAS traces vs reference CaF_2.

Here we report the 200 ns TAS of the BG excitation vs. the reference, just CaF_2.

As clear, the CaF_2 shows a very small signal.

[*]To whom correspondence should be addressed
[†]Department of Chemistry - Ångström Laboratory
[‡]These authors contributed equally to this work.
[¶]Center of Molecular Devices, KTH

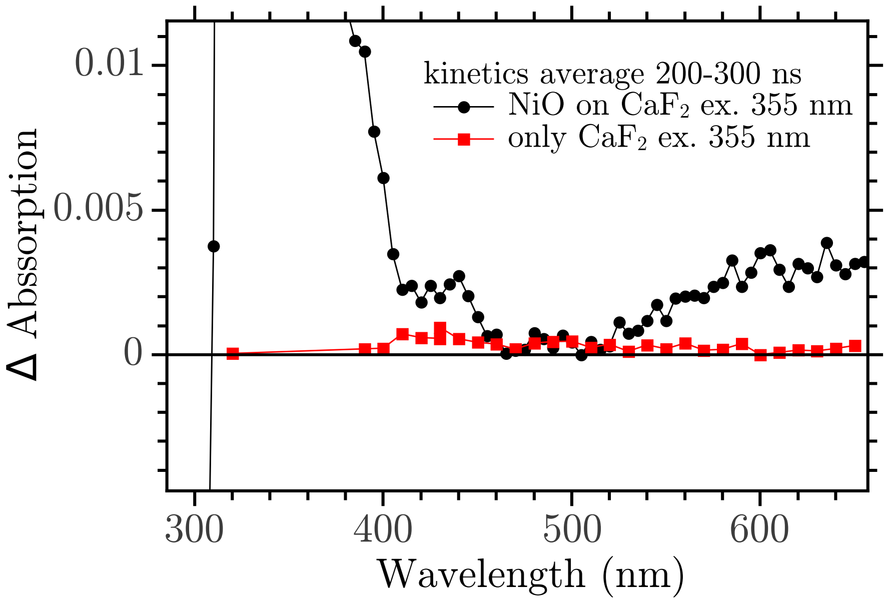

Figure S1: Comparison between the Δ-Abs at 200 ns after BG excitation of the NiO film on CaF_2 and only the CaF_2.

ns TAS traces by excitation at 266 nm

To confirm that the BG excitation occurs really in the BG an excitation with more powerful photon was made and the ns TAS are compared in Figure S2.

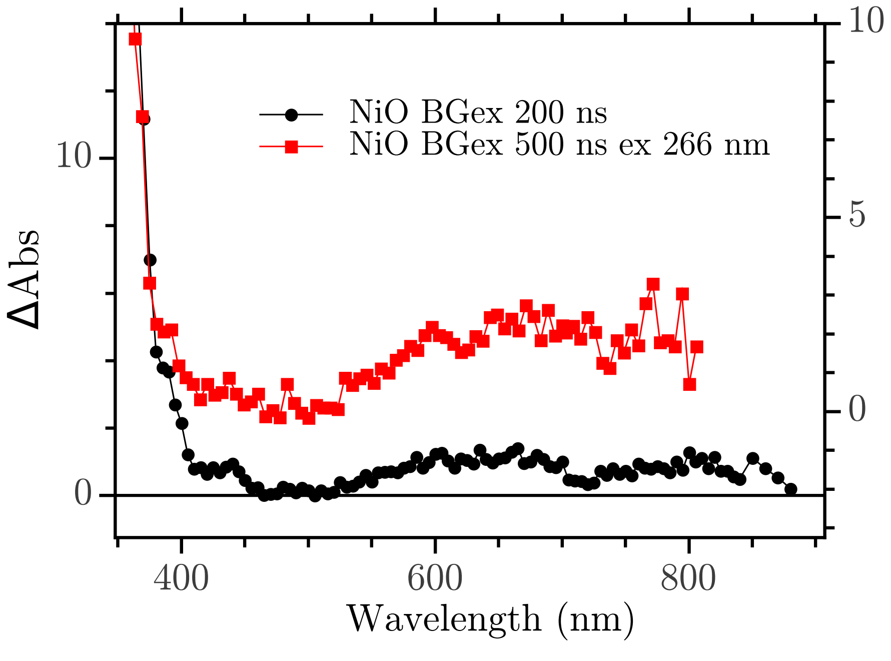

Figure S2: Comparison between the Δ-Abs at 200 ns after BG excitation of the NiO film with 355 nm light and 266 nm light (4 mJ/pulse).

Heating effect on the absorption spectrum

In Figure S3 we report the Δ-Abs of NiO after BG excitation and the difference spectrum betweenthe absorption spectrum recorded at RT and the one at 180°.

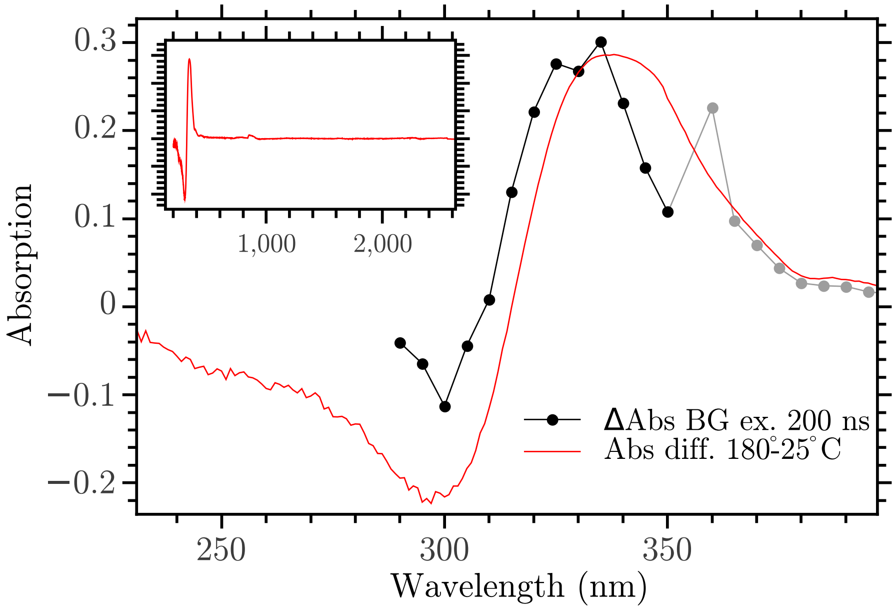

Figure S3: Comparison between the Δ-Abs at 200 ns after BG ex. and the difference between the NiO absorption spectrum at RT and the one at 180°.

Further fs-TAS data

In this section we present the further fs data that were recorded.

fs-TAS with Ni^{3+} free NiO

Figure S4 shows the DAS of a BG excitation of a NiO sample that was heated at 200° C. This treatment removes the Ni^{3+} impurities from the NiO nanoparticle surface.

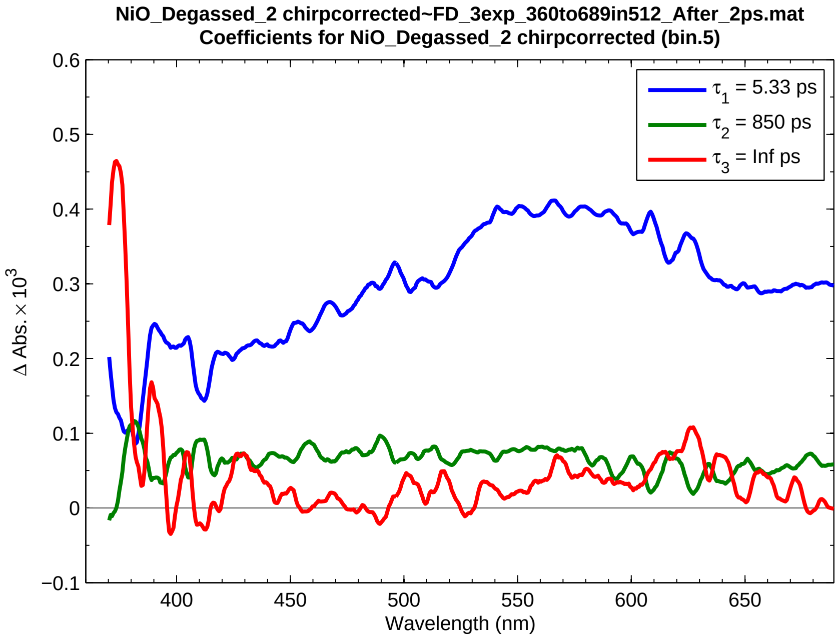

Figure S4: DAS of a BG ex. fs-TAS performed on a NiO film after heating at 200°.

The features that were assigned to Ni^{4+} and Ni^{3+} are not present in the DAS any more. Only a broad band in the red is present that is assigned to the direct electron-hole recombination from conduction band to valence band.

fs-TAS raw spectra and kinetic traces

In Figure S5 the raw spectra recorded in the fs-TAS experiment are reported. The delays ranged from 500 fs to 1.9 ns and are indicated in the plot. In Figure S6 the kinetic traces of specific wavelengths are shown.

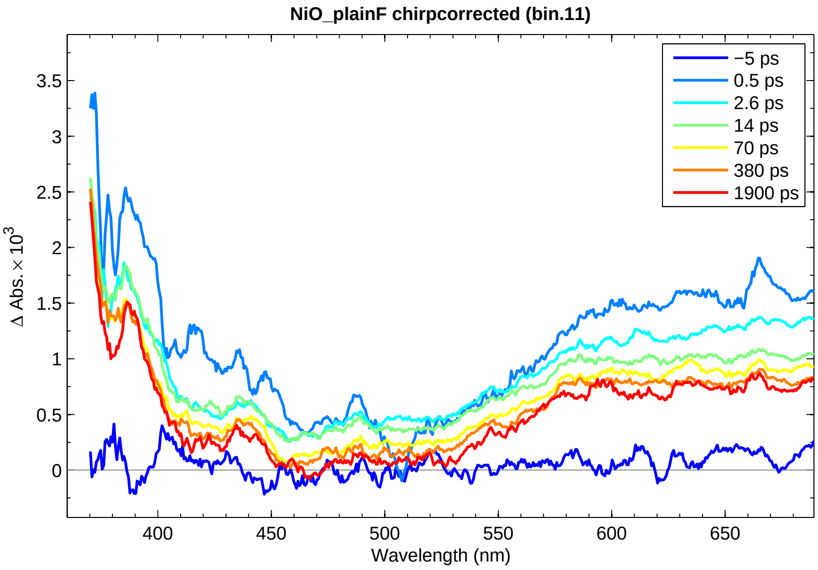

Figure S5: Raw TAS of a BG ex. fs-TAS performed on a NiO film.

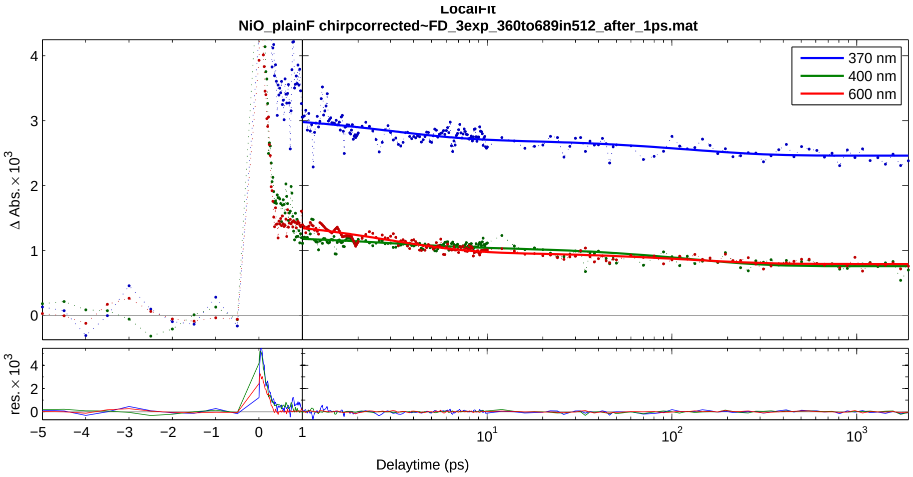

Figure S6: Kinetic traces with fitting of the raw TAS presented in Figure S5.

In Figures S7 and S8 the raw TAS and the kinetic of the Ni^{3+} purified sample traces are reported.

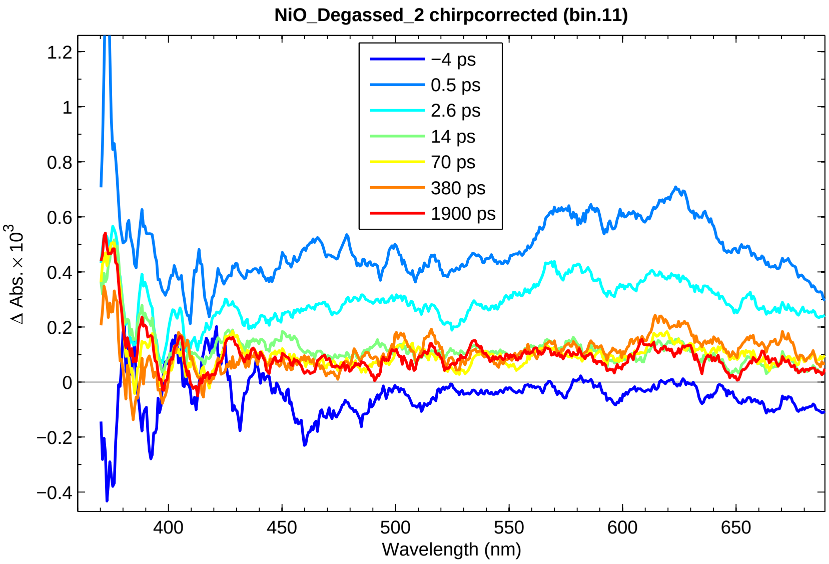

Figure S7: Raw TAS of a BG ex. fs-TAS performed on a NiO film after heating at 200°.

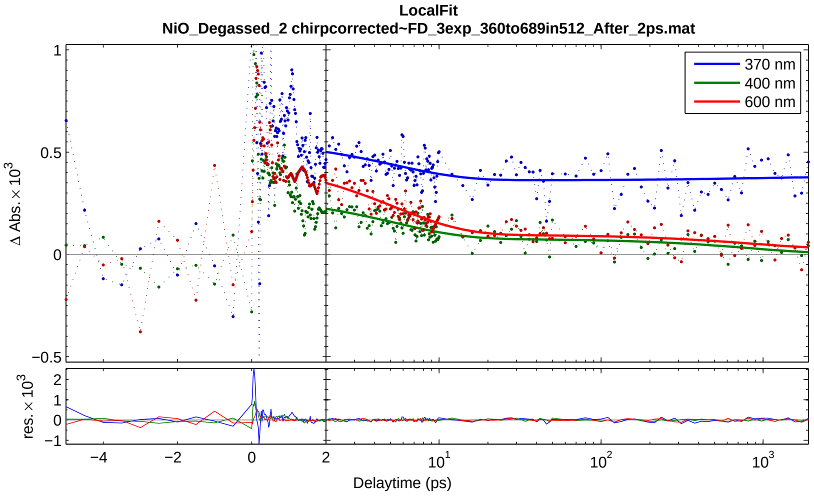

Figure S8: Kinetic traces with fitting of the raw TAS presented in Figure S7.

NiO Band Gap excitation in presence of electron acceptor

The reactivity of the electron in the CB of NiO was tested performing a band gap excitation to a film in contact with an electron scavenger. A saturated solution of methyl viologen was used with this scope. The solution was kept in contact with the film by mean of a microscope cover glass.

In Figure S9 the TA of the wet film recorded 500 ns after excitation is compared with the reference bare NiO TA.

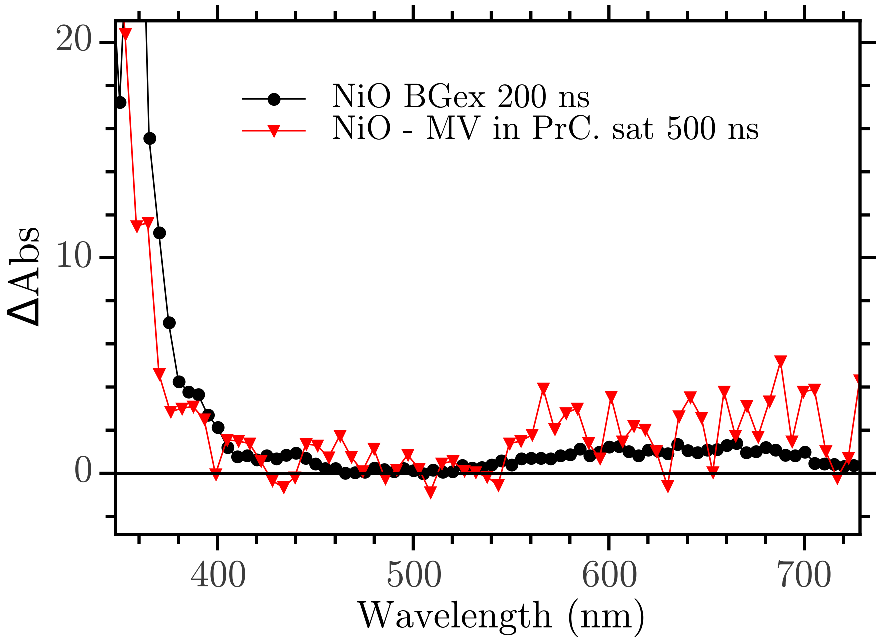

Figure S9: Comparison of the TA of bare NiO and NiO wet by a saturated solution of methyl viologen. The TA of the bare NiO is the result of the averaging of thh kinetic traces in the range 150-250 ns after excitation; instead the NiO-MV^{2+} TA is collected with a CCD camera at 500 ns after excitation (gate width 1000 ns).

The NiO-MV^{2+} TA presents the same features of the bare NiO TA. There was no trace at any time of the $MV^{\cdot+}$ radical which should show a pronounced absorption at 380-400 nm and a broad band around 650 nm. This can be a sign that the electron in the conduction band has been deactivated by falling in a deep trap.

On the kinetic mechanism of electron-hole recombination

In the following the monochromatic traces of the main Δ-Absare reported.

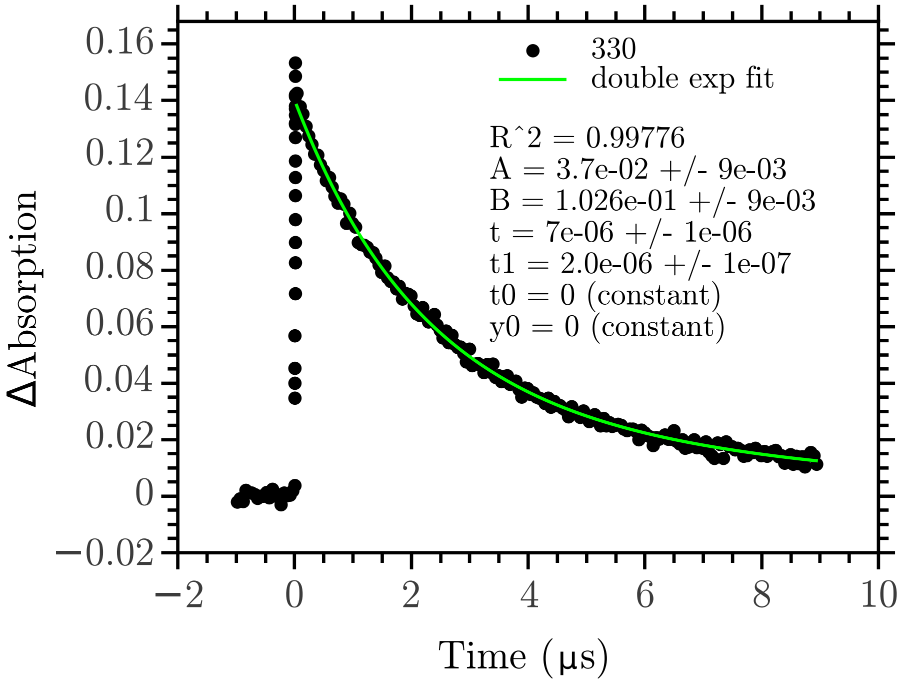

Figure S10: Monochromatic traces recorded after gap excitation of NiO. Trace 330 nm with respective multi exponential fitting.

In the following plot the comparison of the 430 nm band and the 650 nm one is presented.

The traces show a similar kinetics. The 430 nm is now fit with a second order kinetics. As discussed in the main article, the 430 nm trace shows the kinetics of the electron-hole *trap annihilation*:

$$Ni^{4+}_{(\mathbf{h}^{+})} + Ni^{2+}_{(\mathbf{e}^{-})} \longrightarrow Ni^{3+} + Ni^{3+} \tag{1}$$

This reaction can either be a second order reaction or a first order reaction. If the two reactants can freely diffuse on the surface of NiO, this reaction obeys to diffusion law so it is a second order (surface) reaction. On the contrary if the hole and electron trap are bound electrostatically one another and form a pre associated complex the reaction

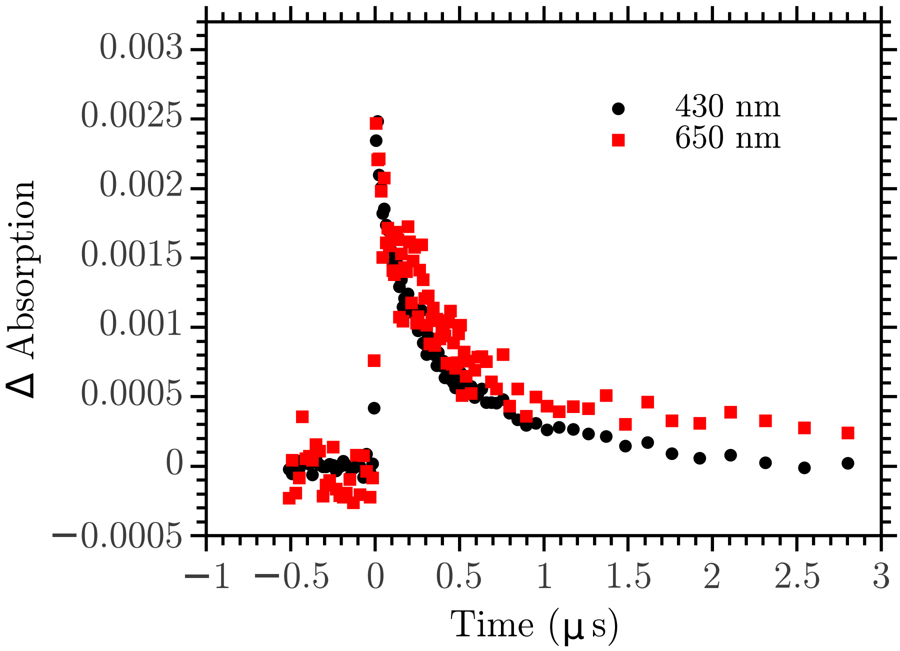

Figure S11: Monochromatic traces recorded after gap excitation of NiO. Traces 430 nm (black) and 650 nm (red)

would be of the first order.

The second order kinetics should be supported by the evidence of initial concentration rate dependence. In other words the reaction should be faster at greater initial concentration of reactants that in this case means higher excitation light intensities. A study was done to confirm this and the results for the 430 nm trace is reported in figure S12. It seems that the kinetics is faster at higher intensities, thus the kinetics of the *trap annihilation* is believed to be of the second order.

In Figure S13 the inverse of the Δ-Absis plotted versus time and fit with a straight line. The fitting does not follow completely the data set. This could be due to several factor. First of all the amplitude of these decays are very small considering the nature of the sample (film). Secondly the local temperature of the film is changing of several decades of degree Celsius which can speed up the kinetics in the initial part of the decay. Thirdly the presence of Ni^{2+} was not consider affecting the kinetics while it could have an effect, considering the set of reactions for example:

Hole relaxation to a $Ni^{3+}_{(\mathbf{h}^+)}$ trap:

$$Ni^{4+}_{(\mathbf{h}^+)} + Ni^{2+} \longrightarrow Ni^{3+} + Ni^{3+}_{(\mathbf{h}^+)} \tag{2}$$

Hole transport through the Ni^{2+} or Ni^{3+} net:

$$Ni^{3+}_{(\mathbf{h}^+)} + Ni^{3+} \longrightarrow Ni^{3+} + Ni^{3+}_{(\mathbf{h}^+)} \tag{3}$$

$$Ni^{3+}_{(\mathbf{h}^+)} + Ni^{2+} \longrightarrow Ni^{2+} + Ni^{3+}_{(\mathbf{h}^+)} \tag{4}$$

And finally the hole-electron *trap annihilation*:

$$Ni^{3+}_{(\mathbf{h}^+)} + Ni^{2+}_{(\mathbf{e}^-)} \longrightarrow Ni^{2+} + Ni^{3+} \tag{5}$$

This mechanism take in consideration the Ni^{2+} states that were not created from an electron trapping, i.e. that were already existing in the surface of NiO. These states can work as a hole carrier, as $Ni^{3+}_{(\mathbf{h}^+)}$, transporting the hole trap to to the electron trap $Ni^{2+}_{(\mathbf{e}^-)}$ where they can finally recombine. This mechanism would complicate quite much the kinetics. In this case the Ni^{4+} spectrum vanishes in the first reaction that is a second order reaction with two different starting concentration. From our data it seems that this is not the case since the linear plot in Figure S13 and the light intensity dependence shown in Figure S12. Further experiment should be made to verify this hypothesis.

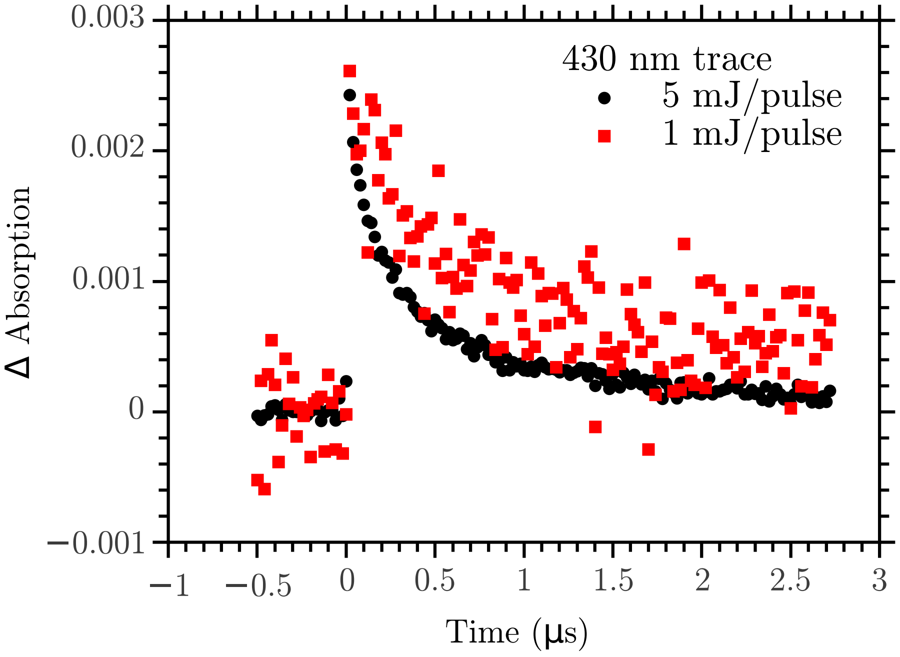

Figure S12: Monochromatic 430 nm traces recorded after gap excitation of NiO at two different excitation light intensities.

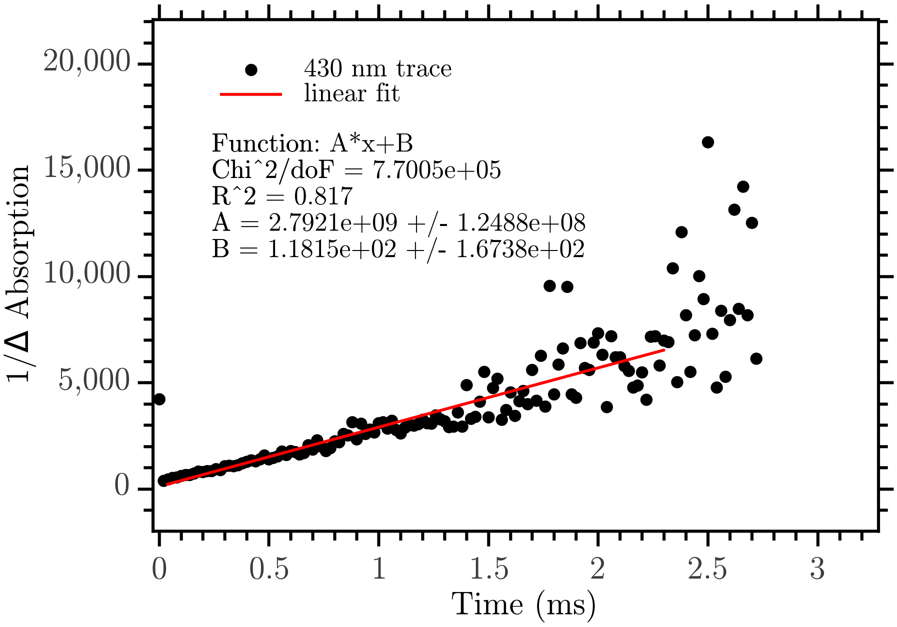

Figure S13: Second order fit of the 430 nm trace.
